# Separase Phosphosite Mutation Leads to Genome Instability and Primordial Germ Cell Depletion during Oogenesis

**DOI:** 10.1371/journal.pone.0018763

**Published:** 2011-04-11

**Authors:** Juan Xu, Meizhi Wang, Xinxing Gao, Bian Hu, Yinan Du, Jiankui Zhou, Xuemei Tian, Xingxu Huang

**Affiliations:** 1 Model Animal Research Center, Nanjing University, Nanjing, China; 2 Department of Anatomy, Histology and Embryology, Southern Medical University, Guangzhou, China; 3 School of Life Science, South China Normal University, Guangzhou, China; Temasek Life Sciences Laboratory, Singapore

## Abstract

To ensure equal chromosome segregation and the stability of the genome during cell division, Separase is strictly regulated primarily by Securin binding and inhibitory phosphorylation. By generating a mouse model that contained a mutation to the inhibitory phosphosite of Separase, we demonstrated that mice of both sexes are infertile. We showed that Separase deregulation leads to chromosome mis-segregation, genome instability, and eventually apoptosis of primordial germ cells (PGCs) during embryonic oogenesis. Although the PGCs of mutant male mice were completely depleted, a population of PGCs from mutant females survived Separase deregulation. The surviving PGCs completed oogenesis but produced deficient initial follicles. These results indicate a sexual dimorphism effect on PGCs from Separase deregulation, which may be correlated with a gender-specific discrepancy of Securin. Our results reveal that Separase phospho-regulation is critical for genome stability in oogenesis. Furthermore, we provided the first evidence of a pre-zygotic mitotic chromosome segregation error resulting from Separase deregulation, whose sex-specific differences may be a reason for the sexual dimorphism of aneuploidy in gametogenesis.

## Introduction

The most fundamental feature of mitotic and meiotic cell division is the equal transmission of the duplicated genome into two daughter cells. This is achieved by the separation of sister chromatids, a process mainly executed by a cysteine protease called Separase [1], [2]. This process occurs with high fidelity by accurate intrinsic chromosome segregation machinery and the activity of a spindle assembly checkpoint [3]. However, errors during cell division do occur, resulting in chromosome instability and leading to aneuploidy, a condition with an abnormal number of chromosomes. Aneuploidy is considered to be the leading genetic cause of human fertility failure. Approximately 10–30% of human zygotes and 50% of spontaneously aborted fetuses have an abnormal number of chromosomes [4]. Genetic etiology studies revealed that the aneuploidy mainly originated from gametogenesis and early embryogenesis during development [5]. Gametogenesis starts from primordial germ cells (PGCs), which undergo mitotic and meiotic cell division to generate gametes with a diploid or a haploid karyotype [6], [7].

It is now generally accepted that the main cytogenetic events that lead to chromosome segregation errors are non-disjunction in meiosis I (MI), premature chromosome segregation in meiosis II (MII) [8], [9] and post-zygotic mitosis [5]. Yet, the molecular mechanisms underlying chromosome segregation errors remain unclear due to the lack of appropriate model systems [10]. There have been several recent successful analyses using mouse models, which demonstrated that the abnormalities in the connections between homologous chromosomes and sister chromatids and related events, including pairing, synapsis, and recombination, can yield chromosome segregation errors [11]–[15]. Moreover, it has been reported that deletion of the meiosis-specific Cohesin component SMC1β causes meiotic chromosome mis-segregation [12], [13]. Cohesin is a key molecular link between female aging and chromosome mis-segregation during MI [16]. Separase functions in arm cohesion dissociation, chiasma resolution, and meiosis I exit [17], [18]. These data strongly suggest that Separase deregulation may cause chromosome segregation errors during gamatogenesis.

To ensure proper chromosome segregation, Separase is strictly controlled by two elaborate mechanisms in mammals. First, Separase is inhibited through association with Securin, which is degraded by the proteasome after anaphase-promoting complex (APC)-mediated polyubiquitination at the metaphase/anaphase transition [19]–[23]. Second, inhibitory phosphorylation of Ser1126 and Thr1326 (Ser1121 and Thr1321 in the mouse, respectively) of Separase [24], which allow binding with Cyclin B1 and result in the inhibition of Separase activity [25]. In addition, Clift and colleagues demonstrated that Shugoshin can prevent Separase activation independently of Securin [26]. Sun and coworkers found that Cohesin cleavage by Separase was bridged by DNA in a sequence-nonspecific manner [27].

By generating a knock-in mouse model expressing non-phosphorylatable Separase with a S1121A point mutation, we have explored the Separase phospho-regulation at organismal level. We observed that both male and female mutant mice are infertile. We first determined that the sterility of males arose from the failure of spermatogenesis caused by exclusively depletion of PGCs in which Securin was expressed at low levels. This meant that the inhibition of Separase in PGCs was dependent mainly on inhibitory phosphorylation [28]. Upon further investigation of the female infertility, we uncovered that the Separase phosphosite mutation leads to reduced folliculogenesis to give birth to deficient initial follicles, which results from partial depletion of female PGC. Furthermore, we defined a gender-specific discrepancy of Securin, which may explain the sexual dimorphism of the Separase mutation-mediated PGC defects.

## Results

### Separase S1121A point mutation resulted in deficient initial follicles, decreased ovulation and less fertilization

**Figure 1:**
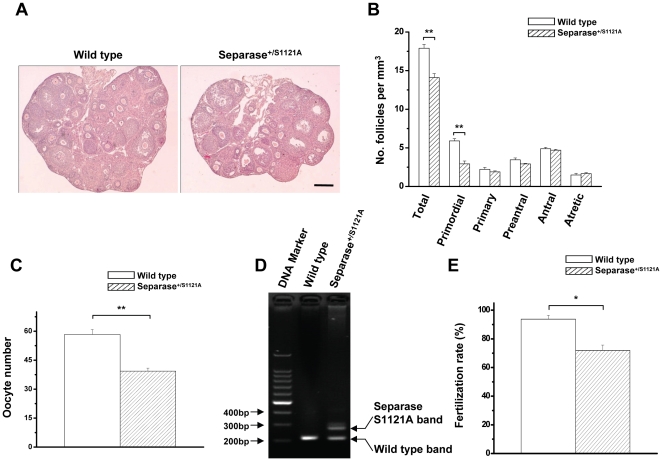
Reduced follicles, decreased ovulation and decreased fertilization of female Meox-2-cre/Separase S1121A mutant mice. A. Hematoxylin-eosin staining of ovary sections obtained from three-week-old mice. Bar = 5.0 µm. B. Quantitative analysis of different kinds of follicles of three-week-old mice (n = 3) (** p<0.001, T test). C. Quantitative analysis of super-ovulation of four-week-old mice (n = 5) (** p<0.001, T test). D. Genotyping of superovulated oocytes from four-week-old wild-type and heterozygous Separase^S1121A^ mice. Two bands are visible. The lower bands in both lanes are the wild-type Separase allele; the upper band is the Separase^S1121A^ allele. E. Quantitative analysis of the fertilization rate of four-week-old mice (n = 4) (** p<0.001, T test).

We recently reported our initial observations of mice carrying a non-phosphorylable Separase allele [28], [29]. The crosses between Separase^+/S1121A-Puro^ mice and Meox2^+/Cre^ mice yielded mutants carrying a point mutation in Separase, Meox2^+/Cre^/Separase^+/S1121A^. Mating analysis revealed that, in contrast to their normal fertile littermates, including Meox2^+/+^Separase^+/+^ (wild type), Meox2^+/+^/Separase^+/S1121A-flox-Puro^, or Meox2^+/Cre^/Separase^+/+^, both male and female Meox2^+/Cre^/Separase^+/S1121A^ mice (hereafter named mutant) were sterile. Previously, we determined that the infertility of mutant males was due to spermatogenesis failure characterized by smaller testes and lack of gametes [28]. To our surprise, the ovaries of mutant female mice developed without apparent abnormalities (Figure S1). Also, the mutant oocytes developed into different kinds of follicles, including primordial, primary, preantral, and antral follicles, and they produced atretic follicles and corpora lutea as shown in Figure 1A. These results suggested that female mutant mice had normal folliculogenesis. Nevertheless, quantitative assay indicated that the three-week-old mutant mice were deficient in initial follicle numbers (Figure 1B).

We therefore assessed the ovulation of the Separase point mutation mice. We induced superovulation with exogenous gonadotropins in immature Separase control and mutant mice at 3 weeks of age to evaluate the oocyte production of each female. The results showed that mutant mice produced fewer oocytes (39.33±1.53) compared to those of control mice (58.33±2.52) (Figure 1C), indicating that the mutant mice ovulated fewer eggs. Genotyping PCR demonstrated that eggs from mutant mice contained the Separase mutation (Figure 1D). We then performed a fertilization assay. Consistently, Separase mutant mice had a lower fertilization rate (71.94±3.59) than control mice (93.77±2.67) (Figure 1E). Subsequently, the mutant ovaries degenerated (Figure S1), and mutant follicles exhausted faster from aging than those of control mice (data not shown).

It is noteworthy that the fertilization success rate of the mutants was lower than that of the controls, suggesting there may be some defects in the mutant oocytes that affected their fertilization. In addition, although the mutant mice displayed normal folliculogenesis, and the mutant oocytes were capable of being fertilized, mutant mice were unable to bear young because of the critical role the phosphorylation of Separase plays in early embryogenesis, when Securin expression is very low [29].

### The Separase S1121A point mutation led to the partial depletion of female PGCs

**Figure 2:**
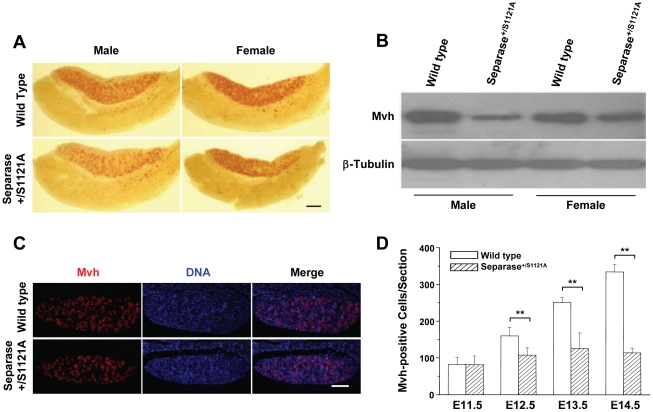
Partial depletion of PGCs in female Meox-2-cre/Separase S1121A mutant mice. A. Whole-mount TNAP staining of female gonads of 13.0 dpc. TNAP-positive cells are red. Bar = 20.0 µm. B. Western blot detection of Mvh of gonads at 13.0 dpc. C. Mvh immuno-staining of gonads at 13.0 dpc. Mvh-positive cells are red. Bar = 4.0 µm. D. Quantitative analysis of Mvh-positive cells in female gonads of different stages. Serial sections of female genital ridge were stained for DNA and Mvh. The Mvh-positive cells were scored from at least three sections in at least three embryos (at least six genital ridges) for each time point. The mean value is shown with standard error (** p<0.001, T test).

Folliculogenesis is a process succeeding oogenesis; like spermatogenesis, it starts from ∼45 PGCs around 7.0 days post-conception (dpc) during embryogenesis [7]. We followed the female development over time. Using PGC-specific, tissue non-specific alkaline phosphatase (TNAP) staining, we found that both control and mutant E11.5 genital ridges of the female were stained identically to that of the male (data not shown). As development progressed, fewer red TNAP-positive cells remained in mutant female gonads compared to those of controls. However, compared to mutant male samples, more TNAP-positive cells were present in mutant female genital ridges (Figure 2A). We further examined the situation by Western blot and immunostaining with mouse vasa homologue (Mvh), another PGC-specific marker. As shown in Figure 2B, both male and female mutant genital ridges expressed less Mvh than their control, but the decrease of Mvh level in the female mutant was significantly less than that in the male mutant. *In situ* immunostaining showed that many Mvh-positive cells remained in female gonads (Figure 2C & D). These results indicated that, in contrast to the complete depletion of PGCs seen in male mutant mice [28], the Separase mutation only partially impaired the development of female PGC, resulting in deficient initial follicles but not in complete PGC depletion.

### Separase S1121A point mutation induced aberrant mitosis and mitotic arrest of female PGCs

**Figure 3:**
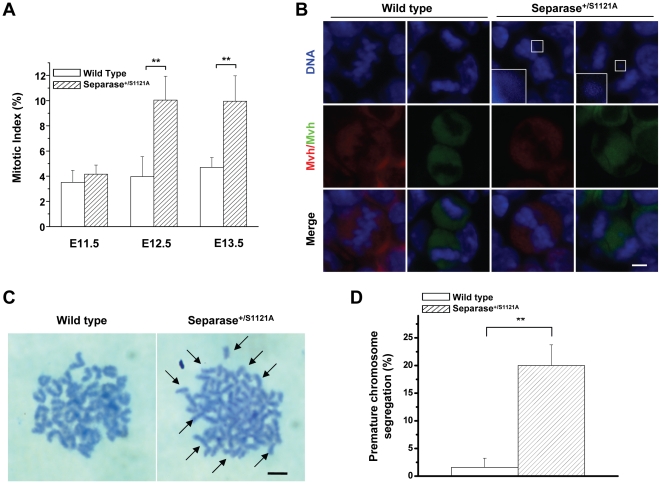
Abnormal mitotic chromosome segregation in PGCs of female Meox-2-cre/Separase S1121A mutant mice. A. The graph shows the calculated mitotic indices of the female PGCs from wild-type and Separase^S1121A^ gonads of various ages as indicated (n = 3) (** p<0.001, T test). B. Aberrant mitotic configurations of female PGCs at 13.0 dpc detected by immuno-staining with Mvh (green/red) and DNA (blue). Inset: higher magnification of DAPI-stained misaligned chromosome and lagging chromosome. Bar = 0.5 µm. C. Chromosome spreads of cultured female PGCs at 13.0 dpc after 6 h nocodazole treatment. Arrows indicated the prematurely separated sister chromatids. Bar = 1.0 µm. D. Quantitative analysis of premature chromosome segregation from chromosome spreads of cultured female PGCs at 13.0 dpc after 6 h nocodazole treatment (n = 4) (** p<0.001, T test).

We found that Separase point mutation led to developmental defects in male PGCs, which exhibited mitotic arrest resulting from premature chromosome segregation and an aberrant mitosis-activated spindle assembly checkpoint [28]. This suggests that developmental defects induced by Separase point mutation in female mutant PGCs may also be the consequences of mitotic errors and mitotic arrest. Therefore, mitosis in the PGCs of female mutant mice was carefully observed by immunostaining for DNA, Mvh, and phospho-Histone 3, one of the mitosis markers. As expected, normal mitotic cell configurations in the different mitosis stages were visualized (data not shown). The female PGC mitotic arrest was evidenced by quantitative evaluation showing that the mitotic indices of mutant PGCs increased slightly at 11.5 dpc and became significantly higher than that of control PGCs from 12.5 dpc to 13.5 dpc (Figure 3A). Similarly, the mitotic arrest was accompanied by aberrant mitosis characterized by mis-aligned and lagging chromosomes (Figure 3B), which are the typical mitotic errors resulting from premature chromosome segregation. Furthermore, sister chromatids remained intact in the control PGCs but prematurely separated in the mutant PGCs (Figure 3C & D).

Like the mutant male PGCs, the mutant female PGCs with abnormal nuclei adapted to sustained mitotic checkpoint activation featured with abnormal Aurora B staining [28] (Figures S2A & B).

### Separase S1121A point mutation caused genome instability of mutant female PGCs

**Figure 4:**
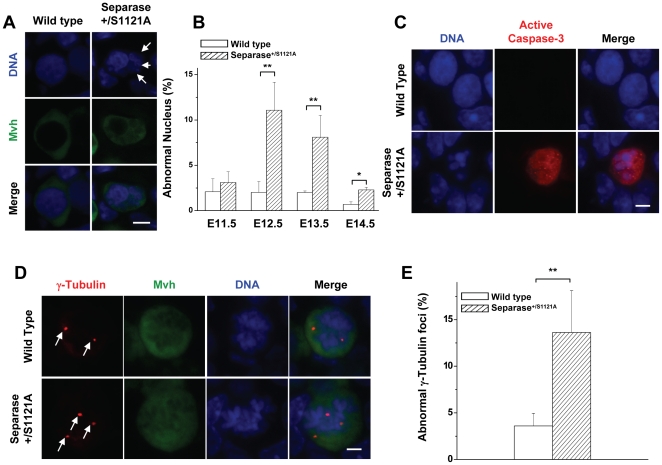
Genome instability in PGCs of female Meox-2-cre/Separase S1121A mutant mice. A. Micronucleus configuration of female PGCs at 13.0 dpc observed by immuno-staining with Mvh (green) and DNA (blue). Arrows indicate the micronuclei. Bar = 0.5 µm. B. Quantitative analysis of micronuclei of female PGCs from wild-type and Separase^S1121A^ gonads of various ages as indicated. Serial sections of female genital ridges were stained for DNA and Mvh. All the Mvh-positive cells and the Mvh-positive cells with micronuclei were scored from at least three sections in at least three embryos (six genital ridges) for each time point. The mean value is shown with standard error (** p<0.001, T test). C. Representative of apoptotic PGCs detected by active Caspase-3 (red)-positive PGC with abnormal nucleus (blue) configuration. Bar = 2.0 µm. D. Centrosome number in female PGCs visualized by immuno-staining with γ-Tubulin (red), Mvh (green) and DNA (blue). Red dots (arrows) of γ-Tubulin staining indicate centrosomes. Bar = 0.5 µm. E. Quantitative analysis of abnormal centrosomes of female PGCs at 13.0 dpc. Serial sections of female genital ridges were stained for DNA, Mvh, and γ-Tubulin. The Mvh-positive cells with three or more γ-Tubulin foci were scored from at least three sections in at least three embryos (six genital ridges). The mean value is shown with standard error (** p<0.001, T test).

It has been previously described that Securin^−/−^/Separase^+/S1121A^ double mutant ES cells [30], Hela cells over-expressing human S1126A mutant Separase [31], and male Separase S1121A mutant PGCs undergo aberrant mitosis and mitotic arrest, resulting in aneuploidy [28]. These facts led us to test whether surviving female PGCs with the Separase point mutation manifested genome instability. PGC nuclei were carefully checked by immunostaining for DNA and Mvh. Micronuclei appeared in female mutant PGCs (Figure 4A). Quantitative evaluation of E11.5 to E14.5 samples showed that the proportion of mutant PGCs with micronuclei increased slightly at 11.5 dpc, then dramatically from 12.5 to 14.5 dpc. Meanwhile, all control samples had few PGCs with abnormal nuclei (Figure 4B), underscoring that it was the Separase point mutation that led to the genome instability of female PGCs, as in male PGCs. Examination of these PGCs by immunostaining for γ-Tubulin revealed that many of them contained multiple centrosomes (Figure 4D & E), confirming the genome instability of the female PGCs. Interestingly, Separase is also involved with the regulation of centrosome duplication [32]. Therefore, multiple centrosomes and abnormal nuclei may be the results of chromosome mis-segregation, or abnormal centrosome duplication, potentially mediated by the deregulation of Separase. The mutant female PGCs underwent apoptosis in the same way as male mutant PGCs, as shown by caspase-3 activation (Figure 4C).

Upon finishing mitotic proliferation, PGCs entered a premeiotic stage. In the male genital ridge, meiosis proceeded no further, and the germ cells entered mitotic arrest as G0/G1 prospermatogonia. In the female genital ridge, however, the germ cells committed to meiosis and passed through leptotene, zygotene, and pachytene stages before arresting in diplotene around the time of birth [7]. We were very interested in the meiotic situation of the mutant female PGCs that bear the Separase mutation. Examination by immunostaining with the meiotic-specific marker phospho-H2AX-γ showed that some of the Mvh-positive cells were co-stained by phospho-H2AX-γ in both female control and mutant PGCs of E13.5 (Figure S3A). There was no significant difference in the percentage of phospho-H2AX-γ-positive cells over Mvh-positive cells (Figure S3B), indicating that the PGCs suffering from Separase mutation have developed into normal meiotic oocytes.

### Sex-specific differences in Securin levels

**Figure 5:**
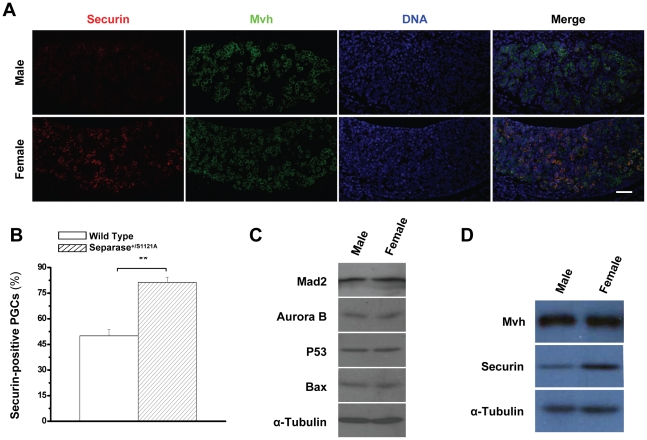
More Securin expression in female PGCs. A. Securin levels of PGCs at 13.0 dpc observed by immuno-staining with Securin (red), Mvh (green) and DNA (blue). Bar = 5.0 µm. B. Quantitative analysis of Securin-positive PGCs in male and female gonads at 13.0 dpc. Serial sections of female genital ridges were stained for DNA, Mvh, and Securin. All Mvh-positive cells and the Mvh and Securin double positive cells were scored from at least three sections in at least three embryos (six genital ridges) for each time point. The mean value is shown with standard error (** p<0.001, T test). C. Western blot detection of Mad2, Aurora B, P53, and Bax of gonads at 13.0 dpc. D. Western blot detection of Mvh and Securin of gonads at 13.0 dpc.

Although the Separase mutation induced similar mitotic defect in male and female PGCs, the above results indicated that there was an apparent sex-specific difference in the efficiency of abnormal PGC depletion. Similar to the meiotic aneuploidy, this discrepancy may be due to the fact that the checkpoint functions more effectively in spermatogenesis than in oogenesis [33], [34]. Thus, we postulated that the gender effects of Separase mutation-mediated mitotic defects could be due to a relaxed mitotic checkpoint. To test this possibility, we carefully checked the mitotic checkpoint regulators, Aurora B and Mad2, and the downstream effectors, P53 and Bax, which played crucial roles in Separase mutation-mediated male PGC depletion [28]. The results failed to prove our hypothesis, as both male and female gonads had equal level of the mitotic regulators and effectors (Figure 5C), suggesting there may be other mechanisms involved in the gender discrepancy of Separase mutation-mediated defects.

Because Securin and inhibitory phosphorylation act redundantly to control Separase activity [30], we asked whether there was gender discrepancy in Securin levels. To this end, we checked the levels of Securin in PGCs by *in situ* immunostaining with Securin, Mvh and DNA. Interestingly, we observed that considerably fewer Securin-positive PGCs with low level of Securin were present in male genital ridges than in female (Figure 5A & B). To further confirm these intriguing results, the overall level of Securin in genital ridges was normalized against Mvh. Examination by Western blot showed that there was considerably more Securin in female genital ridges than in male genital ridges (Figure 5D), indicating that there was a sex-specific difference in Securin production of PGCs.

### Securin levels were correlated to Separase S1121A point mutation-mediated genome instability

**Figure 6:**
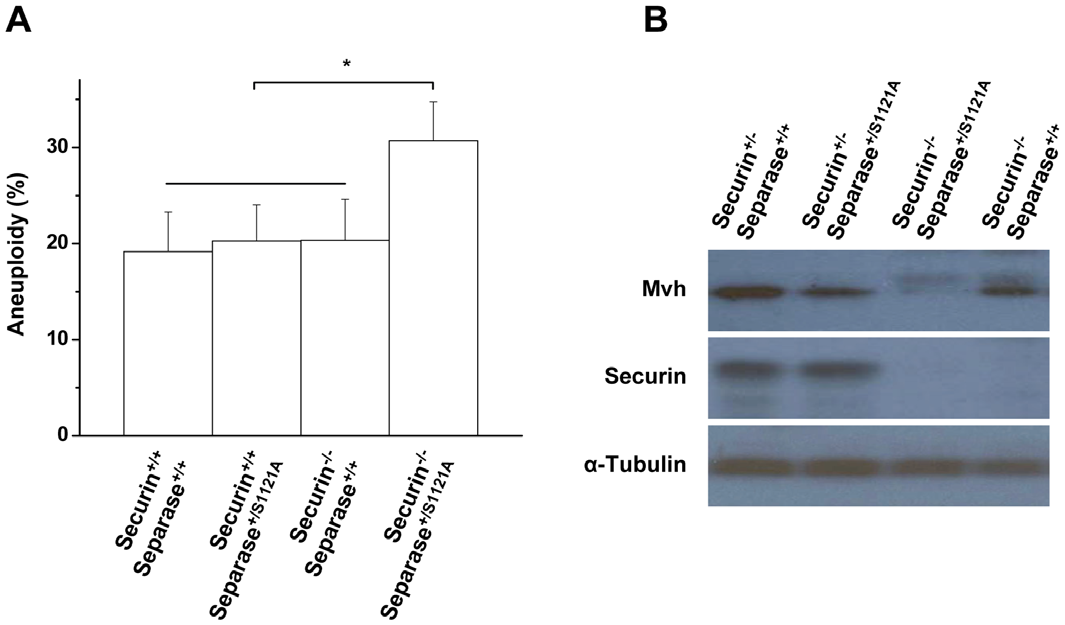
Securin expression is correlated to Separase-mediated defects. A. Quantitative analysis of karyotyping of the four types of ES cells (n = 3) (* p<0.05, T test). B. Western blot detection of Mvh and Securin of urogenital ridges at 10.5 dpc.

We suspected that the sex-specific difference in Securin is correlated with the sexual dimorphism effects of Separase S1121A point mutation on PGCs. We first tried to provide convincing evidence by carefully comparing the phenotype of wild-type, Securin^+/+^/Separase^+/S1121A^, Securin^−/−^/Separase^+/+^, and Securin^−/−^/Separase^+/S1121A^ ES cells. As we observed before, all the cells grew well without aneuploidy [30]. Because double mutant ES cells undergo premature chromosome segregation and grow slower once challenged with one of the spindle poisons, nocodazole [30], we doubted that we would be able to catch the anouploidy of the double mutant cells because of selection against aneuploidy. Therefore, we double-challenged the cells with nocodazole to induce chromosome mis-segregation. Interestingly, the double mutant cells displayed a significantly higher percentage of aneuploidy (30.70±4.06) than wild-type (19.18±4.10), Securin^+/+^/Separase^+/S1121A^ (20.29±3.74), or Securin^−/−^/Separase^+/+^ (20.36±4.25) cells (Figure 6A). These results indicated that the Separase mutation did not cause defects in the presence of Securin in ES cells, but it did lead to chromosome segregation errors in the absence of Securin. Therefore, Securin affected the Separase mutation-mediated defects in ES cells.

By TNAP staining, we demonstrated that the Securin^−/−^/Separase^+/+^, Securin^+/−^/Separase^+/+^, and Securin^+/−^/Separase^+/S1121A^ mice have normal PGCs at 10.5 dpc, while the Securin^−/−^/Separase^+/S1121A^ mice do not, suggesting that Securin expression affected Separase mutation-induced defects on PGCs [28]. To further confirm these results, the overall levels of Mvh and Securin in urogenital ridges of 10.5 dpc were examined by Western blot. Consistently, Separase mutation led to Mvh depletion only in the absence of Securin (Figure 6B). These data suggested that as in ES cells, the Separase mutation did not cause defects in the presence of Securin in PGCs, but it did lead to chromosome segregation errors in the absence of Securin. Therefore, Securin affected the phenotype of Separase S1121A point mutation in PGCs. The sex-specific difference of Securin levels may be correlated to the sexual dimorphism effects of the Separase S1121A point mutation on PGCs.

## Discussion

An important task facing living organisms is the maintenance of the genome stability and its transfer to offspring. Genome transfer is achieved by chromosome segregation. Mis-segregation of chromosomes leads to genome instability [3], [5], [35], [36]. The final executor of chromosome segregation, Separase, is required for development [37], [38]. Moreover, proper regulation of Separase is essential to ensure its proper function in organism genome transfer. Separase is controlled by directly binding to Securin, a reaction that is essential for fission yeast and flies [18], [39]–[41]. Although Securin is dispensable for mammalian cells and mouse [28], [42]–[45], inhibitory phosphorylation provides an additional layer of regulation in vertebrates [24], [25]. By generating a mouse model that expresses a Separase S1121A point mutation, we previously demonstrated that inhibitory phosphorylation is critical for proper Separase regulation and genome stability during spermatogenesis, the gametogenesis process of males [28]. Using this model, we provided evidences in present study that the Separase inhibitory phospho-regulation machinery is also required for proper Separase regulation and genome stability during oogenesis, the gametogenesis process of females.

### Separase phospho-regulation is crucial exclusively for gametogenesis

The genome transfer to offspring is carried out by gametes produced by gametogenesis, including spermatogenesis and oogenesis [3]. Both spermatogenesis and oogenesis start from PGCs, specific cells that are characterized by two rounds of genome segregation performed during mitosis and meiosis, separately [7], which may attribute some specificity to Separase regulation in PGCs. Our observations indicated that in contrast to somatic cells with dually controlled Separase, PGCs have insufficient Securin to fully inhibit Separase and, therefore, their inhibition of Separase is dependent mainly on inhibitory phosphorylation. Without Securin regulation, Separase is well regulated in all kinds of cells *in vivo* [30], [42]–[45], whereas Separase functions poorly in PGCs without phospho-regulation, resulting in the premature segregation of sister chromotids and other chromosome segregation errors, including chromosome misalignment and mis-segregation. These errors lead to genome instability and gametogenesis failure. Our results indicated that Separase phospho-regulation is crucial for gametogenesis and only gametogenesis.

### Separase deregulation in mitosis of gametogenesis is a potential origin of aneuploidy

The vast majority of aneuploidy during gametogenesis comes from meiotic division [5], [10], [34], [46], [47], which produces chromosome segregation errors including non-disjunction in MI and premature chromosome segregation in MII. Our present study provides the first evidence that PGCs undergo mitotic chromosome segregation errors at the beginning of gametogenesis. Therefore, during gametogenesis, chromosome segregation errors come not only from meiosis but also from mitosis.

The meiotic chromosome segregation errors result from abnormalities in the connection between homologous chromosomes and sister chromatids as well as related events such as pairing, synapsis, and recombination [11]–[15]. In contrast, the mitotic chromosome errors we described here occurred due to de-regulation of Separase, the upstream protease of cohesion. We have therefore uncovered a potential origin for aneuploidy during gametogenesis.

### Sexually dimorphic effect of Separase de-regulation on PGCs may be an additional possibility of sex-specific differences in aneuploidy incidences during oogenesis

It is well known that there are sex-specific differences in aneuploidy incidence during gametogenesis. Different possibilities account for the sex-specific differences: first, the differences in the recombination patterns between male and female meiosis; second, more permissive checkpoints in females than males; third, the age-related loss of chromosome cohesion in female gametes; and fourth, the difference in the ability to detect the consequences of meiotic division errors [5], [11]–[15], [34].

We found here that Separase phosphosite mutation leads to genome instability and partial depletion in female PGCs, whereas Separase phosphosite mutation caused genome instability and complete abolishment in male PGCs [28]. The differential depletion of PGCs between males and females indicates that there are obvious sexually dimorphic effects of Separase de-regulation. Combined with the fact that Cohesin, the substrate of Separase, is one of the reasons for sex-specific differences of gamete aneuploidy [12]–[15], we think that sexually dimorphic effects of Separase de-regulation may serve as additional possibilities of sex-specific differences in aneuploidy incidences during gametogenesis.

### Sexually dimorphic effects of Separase de-regulation on PGCs may be correlated with gender-specific Securin expression

After confirming the identical expression levels of mitotic checkpoint regulators and their downstream effectors in male and female genital ridges, we demonstrated that some established factors are not plausible for the gender discrepancy seen in PGC genome instability and depletion. Separase is redundantly controlled by Securin and phosphorylation [30]. Phosphosite mutations caused Separase deregulation exclusively in PGCs [28] and early embryonic cells [29] because they contain insufficient amount of Securin to regulate Separase. Here we provided evidence that the presence or absence of Securin affected the phenotype of Separase mutation in both ES cells and PGCs. Collectively, we believe that Securin expression is correlated to Separase phosphosite mutation-mediated defects in PGCs.

The facts that Securin expression affects Separase mutation-mediated defects, and the existence of gender-specific Securin expression discrepancy, suggested that, the sexually dimorphic effects of Separase de-regulation on PGCs may be correlated with the gender-specific Securin expression. Females had considerably more Securin than males, which may alleviate the phosphosite mutation-mediated Separase malfunction, resulting in fewer chromosome segregation error occurrences and thus less PGC death in females than in males. How is the Securin gender-specific discrepancy formed? As we know, sex determination changes the gene expression of PGCs [7], [48]. Further investigations are required to decide whether sex determination affects Securin expression or not.

## Materials and Methods

### Ethics Statement

Animal studies were carried out in an SPF animal facility accredited by the Association for the Assessment and Accreditation of Laboratory Animal Care, and all animal protocols were approved by the Animal Care and Use Committee of the Model Animal Research Center, the host for the National Resource Center for Mutant Mice in China, Nanjing University.

### Generation of mice with Separase inhibitory phosphosite mutation

Mice with conditional Separase inhibitory phosphosite mutation were generated, genotyped and sex determined as described [30]. For genotyping, 1–2 mm sections of tail tip were dissolved in 0.1 ml of 50 mM Tris (pH 8.0), 100 mM EDTA, 0.5% SDS, and 0.5 mg/ml proteinase K (Roche) solution at 55°C over night with vigorous shaking. The DNA was purified by phenol/chloroform extraction followed by ethanol precipitation and then dissolved in 0.2 ml of TE buffer. Genotyping PCR was carried out using rTaq (Takara). Primers used for the analysis were as follows: for Separase, pz228a: 5′-cct tct cta acc cag gta gg-3′, pz228b: 5′-aag agc tct acc tac ctc agg-3′, and pz228c: 5′-atc gca tcg agc gag cac gta ctc-3′. Pz228a/b amplifies S1121A allele, and pz228b/c S1121A-flox-Puro. Sexing of the embryos was performed with a PCR assay on genomic DNA for the presence or absence of Sry, a gene only present on the Y chromosome [49].

### Histological analysis

Standard histological procedures were followed to prepare fetal gonads and ovaries. In brief, fetal gonads were fixed in 4% paraformaldehyde solution (Sigma), and ovaries were fixed in 10% neutral buffered formalin (Sigma). The specimens were dehydrated through a graded series of ethanol washes, cleared in Histo-Clear, embedded in wax, and sectioned by standard techniques. Sections (4 µm thick) were dewaxed and stained with PAS-hematoxylin or hematoxylin and eosin (H&E).

### Antibodies

Primary antibodies used for immunofluorescence and Western blotting were mouse anti-α-Tubulin (Sigma), mouse anti-β-Tubulin (clone E7; Developmental Studies Hybridoma Bank, University of Iowa, Iowa City, IA), mouse anti-P53 and rabbit anti-Bax (Santa Cruz Biotechnology, Santa Cruz, CA), rabbit anti-active Caspase-3, mouse anti-Aurora B, and rabbit anti-Mad2 (BD Biosciences PharMingen, San Diego, CA), rabbit anti-phospho-Histone H3 (S10) (Cell Signaling Technology, Danvers, MA), mouse anti-γ-Tubulin (Sigma-aldrich, St. Louis, MO), mouse anti-Securin (Novocastra, Newcastle, UK), and mouse anti-Mvh (a kind gift from Dr. Toshiaki Noce, Mitsubishi-Kasei Institute of Life Science, Tokyo, Japan).

The following secondary antibodies were used: Cy3- and FITC-conjugated anti-rabbit IgG and Cy3- and FITC-conjugated anti-mouse antibodies (Jackson ImmunoResearch Laboratories, Inc.).

### Western blot analysis and immunostaining

The genital ridges or urogenital ridges were collected and lysed on ice in lysis buffer (1% NP-40, 1% sodium deoxycholate, 0.1% SDS, 0.15 M sodium chloride, 2 mM EDTA, 0.01 M sodium phosphate, pH 7.2) supplemented with protease inhibitor cocktail (Roche) and 1 mM phenylmethylsulfonyl fluoride. Protein concentration was determined with Bio-Rad DC protein assay (Bio-Rad). Equal amounts of total protein were separated on denaturing polyacrylamide gel and transferred to polyvinylidene difluoride transfer membrane (Bio-Rad). Blots were probed with the indicated primary and appropriate secondary antibodies and detected using ECL chemiluminescence (GE Healthcare).

For immunoassaying, the dewaxed histological sections of the fatal gonads were antigen-retrieved by immersion of grids bearing sections in 0.1 M sodium citrate buffer, pH 6.0, at 95°C for 10 min, permeabilized with 0.4% Triton X-100 in phosphate-buffered saline (PBS) for 10 min at room temperature, covered with blocking solution (5% goat serum in PBS) for 1 h at 37°C, and incubated with the indicated primary antibodies in blocking solution for overnight at 4°C. Primary antibodies were stained with appropriate secondary antibodies for 60 min at 37°C, followed by 4, 6-diamidino-2-phenylindole for 5 min at room temperature. Fluorescence analysis was carried out with a BX51 Olympus fluorescence microscope connected to a DP 20 digital camera (Olympus Corporation, Japan) and using 20×, 40×, or 63× 1.4 numerical aperture oil immersion objectives at room temperature. The images were collected with software DP controller (Olympus Corporation, Japan), and processed with Adobe Photoshop 7.0 (Adobe Systems Incorporated, San Jose, CA).

For the quantitative evaluation of Mvh-positive cells, serial sections of female genital ridges were stained for DNA, Mvh, and phospho-Histone 3. Mvh-positive cells from at least three sections selected by every two sections in at least three embryos (six genital ridges) were scored for each time point. For the quantitative evaluation of mitotic index, serial sections of female genital ridges were stained for DNA, Mvh, and phospho-Histone 3, Mvh single positive cell, as well as Mvh and phospho-Histone 3 double positive cells were scored from at least three sections selected by every two sections in at least three embryos (six genital ridges) for each time point. The mitotic indices of the female PGCs were the percentage of the number of Mvh and phospho-Histone 3 double positive cells divided by the total number of Mvh-positive cells.

### Whole-mount staining of gonads for PGCs

Dissected genital ridges were fixed with 4% paraformaldehyde, washed with deionized water and then stained for alkaline-phosphatase activity with α-naphtyl phosphate (Sigma)/fast red TR (Sigma) solution [28], [50].

### Collection and culture of germ cells and chromosome spreads

Genital ridges at 11.5 dpc were minced and treated with 0.25% Trypsin/EDTA for 30 min at 37°C to dissociate the cells. The resulting cell suspensions were plated on a feeder layer formed by irradiated LIF-expressing SNL fibroblasts in 15% FBS/DMEM for 2 d. The cells were harvested via trypsin digestion after 6 h nocodazole (65 ng/ml) treatment and subjected to AP staining. AP-positive cells were collected through a mouth-pipette. PGC chromosome spread was performed as described [30]. In brief, the collected AP-positive cells were washed once with 1×PBS, treated with hypotonic solution (0.8% sodium citrate) at 37°C for 30 min, and fixed with Carnoy's Fixative (75% methanol, 25% acetic acid) for 3×10 min. The fixative was dropped onto the slides. The dried slides were stained with Giemsa stain (Sigma) for 30 min.

### Culture of mouse ES cells and chromosome spreads

Mouse ES cells were cultured on a feeder layer formed by irradiated LIF-expressing SNL fibroblasts in 15% FBS/DMEM (Gibco) [30]. Chromosome spread of mouse ES cells was performed as for PGC. For ES cell aneuploidy assay, we treated the cells with 65 ng/ml of nocodazole for 6 h, removed the nocodazole for 12 h, and then treated the cells again with nocodazole for 12 h to prepare chromosome drop. The chromosome numbers of the metaphase spreads for each type of the cells were counted.

### Fertilization assay

Female mice were superovulated by intraperitoneal injection of 5 IU of pregnant mare serum gonadotropin (PMSG; Calbiochem) followed 46 h later by intraperitoneal injection of 5 IU of human chorinonic gonadotropin (hCG; Calbiochem), they were then mated immediately with known fertile male mice. Two-cell stage embryos were collected by flushing the oviducts 43–47 h post hCG. The embryos were stained for DNA. The fertilization rates were the percentage of the number of the 2-cell stage embryos divided by the total number of the collected embryos.

### Statistical analysis

Statistical analysis was performed with Excel 2002 (Microsoft, Redmond, Washington, USA) software. The mean value is shown with standard error. Student's t-test was used for the comparison of two independent groups (*p<0.05, **p<0.001).

## Supporting Information

Figure S1 **Photographs of ovaries.** Representative photos of the ovaries from four-week and one-year-old wild-type and Separase^S1121A^ littermates. Arrows indicate the degenerated ovaries of the one-year-old mutant. Bar = 20.0 µm.
(TIF)

Figure S2 **Abnormal Aurora B expression in mutant female PGCs.** A. Aurora B expression in female PGCs at 13.0 dpc observed by immuno-staining with Aurora B (red), Mvh (green) and DNA (blue). Arrows indicate the Aurora B foci. Bar = 0.5 µm. B. Quantitative analysis of Aurora B-positive PGCs with abnormal nuclei from control and mutant female gonads of 13 dpc. Serial sections of female genital ridges were stained for DNA, Mvh, and Aurora B. All Mvh-positive cells with abnormal nuclei and the Mvh and Aurora B double positive cells with abnormal nuclei were scored from at least three sections in at least three embryos (six genital ridges). The mean value is shown with standard error (**p<0.001, T test).
(TIF)

Figure S3 **Mutant female PGCs proceeded to meiotic oocytes normally.** A. Meiosis of female PGCs at 13.5 dpc detected by immuno-staining with phospho-H2AX-γ (green), Mvh (red) and DNA (blue). Bar = 2.0 µm. B. Quantitative analysis of phospho-H2AX-γ-positive female PGCs from wild-type and Separase^S1121A^ female gonads of 13.5 dpc. Serial sections of female genital ridges were stained for DNA, Mvh, and phospho-H2AX-γ. All Mvh-positive cells and the Mvh and phospho-H2AX-γ double positive cells were scored from at least three sections in at least three embryos (six genital ridges). The mean value is shown with standard error.
(TIF)
