# Supplementary figures and images for: Separase Phosphosite Mutation Leads to Genome Instability and Primordial Germ Cell Depletion during Oogenesis
Source: PLoS One. 2011 Apr 11;6(4):e18763. doi: 10.1371/journal.pone.0018763 (PMC3073988; doi:10.1371/journal.pone.0018763)

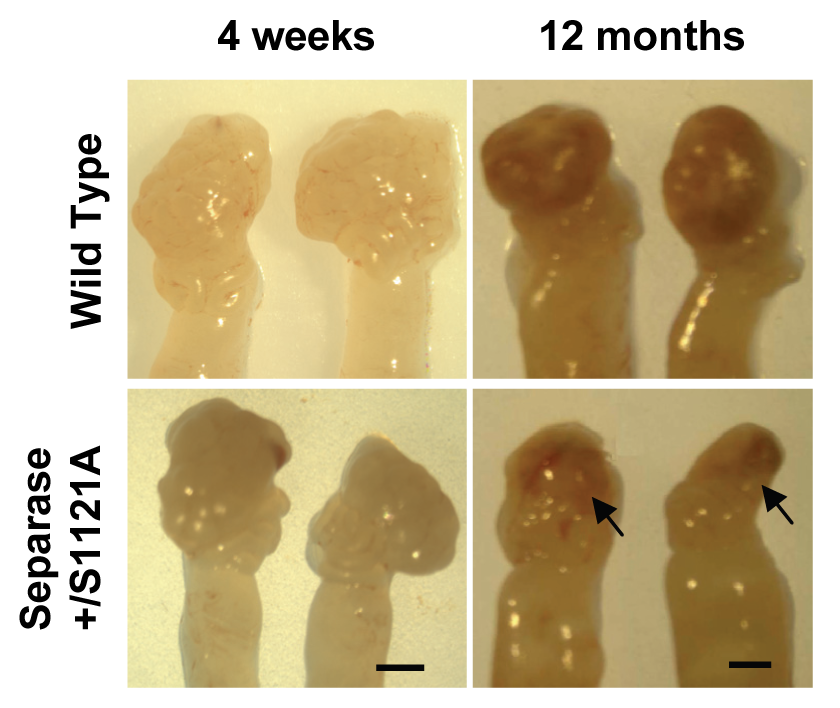

Supplement: Figure S1 — Photographs of ovaries. Representative photos of the ovaries from four-week and one-year-old wild-type and SeparaseS1121A littermates. Arrows indicate the degenerated ovaries of the one-year-old mutant. Bar = 20.0 µm. (TIF) [file pone.0018763.s001.tif]

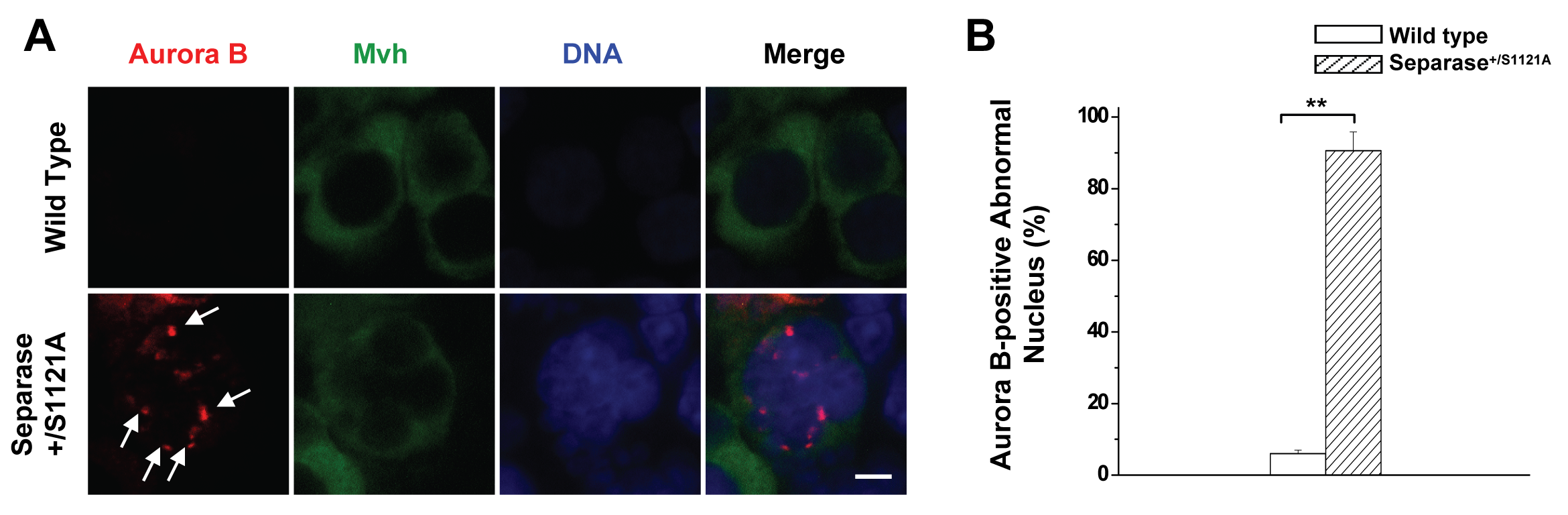

Supplement: Figure S2 — Abnormal Aurora B expression in mutant female PGCs. A. Aurora B expression in female PGCs at 13.0 dpc observed by immuno-staining with Aurora B (red), Mvh (green) and DNA (blue). Arrows indicate the Aurora B foci. Bar = 0.5 µm. B. Quantitative analysis of Aurora B-positive PGCs with abnormal nuclei from control and mutant female gonads of 13 dpc. Serial sections of female genital ridges were stained for DNA, Mvh, and Aurora B. All Mvh-positive cells with abnormal nuclei and the Mvh and Aurora B double positive cells with abnormal nuclei were scored from at least three sections in at least three embryos (six genital ridges). The mean value is shown with standard error (**p<0.001, T test). (TIF) [file pone.0018763.s002.tif]

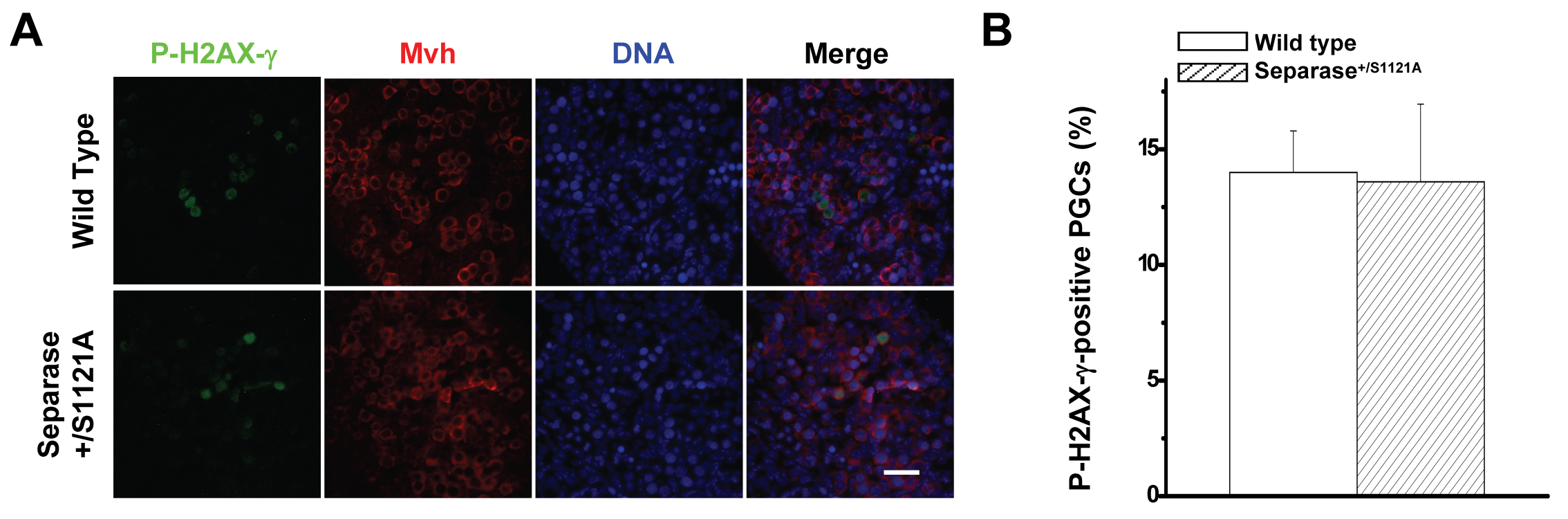

Supplement: Figure S3 — Mutant female PGCs proceeded to meiotic oocytes normally. A. Meiosis of female PGCs at 13.5 dpc detected by immuno-staining with phospho-H2AX-γ (green), Mvh (red) and DNA (blue). Bar = 2.0 µm. B. Quantitative analysis of phospho-H2AX-γ-positive female PGCs from wild-type and SeparaseS1121A female gonads of 13.5 dpc. Serial sections of female genital ridges were stained for DNA, Mvh, and phospho-H2AX-γ. All Mvh-positive cells and the Mvh and phospho-H2AX-γ double positive cells were scored from at least three sections in at least three embryos (six genital ridges). The mean value is shown with standard error. (TIF) [file pone.0018763.s003.tif]
